# Retraction: Psychometric characteristics of the ankylosing spondylitis quality of life questionnaire, short form 36 health survey, and functional assessment of chronic illness therapy-fatigue subscale

**DOI:** 10.1186/1477-7525-7-34

**Published:** 2009-04-17

**Authors:** Dennis A Revicki, Anne M Rentz, Michelle P Luo, Robert L Wong, Lynda C Doward, Stephen P McKenna

**Affiliations:** 1 Center for Health Outcomes Research, United Biosource Corporation, Bethesda, MD, USA; 2 Formerly Abbott Laboratories, Global Health Economics & Outcomes Research, Abbott Park, IL, USA; 3 Abbott Laboratories, Abbott Immunology, Parsippany, NJ, USA; 4 Galen Research, Manchester, Lancashire, England, UK

## Abstract

Retraction of Revicki DA, Rentz AM, Luo MP, Wong RL, Doward LC, McKenna SP: Psychometric characteristics of the ankylosing spondylitis quality of life questionnaire, short form 36 health survey, and functional assessment of chronic illness therapy-fatigue subscale. *Health and Quality of Life Outcomes *2009, **7**: 6.

## Retraction

Not all authors approved of the final content or publication of this article [1]. Subsequently, all authors have agreed that the article should be retracted. This article is being retracted as assumptions were made about the nature of the outcome data that should have been tested before the analyses were conducted. In addition, there is disagreement among the authors about the statistical analyses that were used and it is possible that incorrect conclusions may have been drawn that are not supported by the data presented. The authors wish to apologise for any inconvenience this may have caused the editorial, publishing staff and readers.
